# Repurposing of anti-malarial drugs for the treatment of tuberculosis: realistic strategy or fanciful dead end?

**DOI:** 10.1186/s12936-024-04967-2

**Published:** 2024-05-03

**Authors:** Thomas Hanscheid, Claire Ruiz del Portal Luyten, Sabine M. Hermans, Martin P. Grobusch

**Affiliations:** 1 Instituto de Microbiologia, Faculdade de Medicina, Universidade de Lisboa, Lisbon, Portugal; 2 Center for Tropical Medicine and Travel Medicine, Department of Infectious Diseases, Amsterdam Infection and Immunity, Amsterdam Public Health, Amsterdam UMC, Location University of Amsterdam, Meibergdreef 9, 1105 AZ Amsterdam, Netherlands; 3 Department of Global Health, Amsterdam Institute for Global Health and Development, Amsterdam UMC, Location University of Amsterdam, Amsterdam, Netherlands; 4 Institute of Tropical Medicine, German Centre for Infection Research (DZIF), University of Tübingen, Tübingen, Germany; 5 Centre de Recherches Médicales en Lambaréné (CERMEL), Lambaréné, Gabon; 6 Masanga Medical Research Unit (MMRU), Masanga, Sierra Leone; 7 Institute of Infectious Diseases and Molecular Medicine (IDM), University of Cape Town, Cape Town, South Africa

**Keywords:** Antimalarials, Tuberculosis, Drug re-purposing, In vitro, In vivo, Clinical trials

## Abstract

**Background:**

Drug repurposing offers a strategic alternative to the development of novel compounds, leveraging the known safety and pharmacokinetic profiles of medications, such as linezolid and levofloxacin for tuberculosis (TB). Anti-malarial drugs, including quinolones and artemisinins, are already applied to other diseases and infections and could be promising for TB treatment.

**Methods:**

This review included studies on the activity of anti-malarial drugs, specifically quinolones and artemisinins, against *Mycobacterium tuberculosis* complex (MTC), summarizing results from in vitro, in vivo (animal models) studies, and clinical trials. Studies on drugs not primarily developed for TB (doxycycline, sulfonamides) and any novel developed compounds were excluded. Analysis focused on in vitro activity (minimal inhibitory concentrations), synergistic effects, pre-clinical activity, and clinical trials.

**Results:**

Nineteen studies, including one ongoing Phase 1 clinical trial, were analysed: primarily investigating quinolones like mefloquine and chloroquine, and, to a lesser extent, artemisinins. In vitro findings revealed high MIC values for anti-malarials versus standard TB drugs, suggesting a limited activity. Synergistic effects with anti-TB drugs were modest, with some synergy observed in combinations with isoniazid or pyrazinamide. In vivo animal studies showed limited activity of anti-malarials against MTC, except for one study of the combination of chloroquine with isoniazid.

**Conclusions:**

The repurposing of anti-malarials for TB treatment is limited by high MIC values, poor synergy, and minimal in vivo effects. Concerns about potential toxicity at effective dosages and the risk of antimicrobial resistance, especially where TB and malaria overlap, further question their repurposing. These findings suggest that focusing on novel compounds might be both more beneficial and rewarding.

**Supplementary Information:**

The online version contains supplementary material available at 10.1186/s12936-024-04967-2.

## Background

Drug repurposing, also known as drug repositioning, has emerged as an important strategy in contemporary medicine (Table 1). This approach, distinct from traditional drug discovery methods, leverages the known pharmacokinetics and safety profiles of existing drugs. By doing so, it side-steps the significant financial and time constraints inherent to the development and clinical testing of new compounds [1, 2]. Prime illustrations of this strategy include drugs like aspirin, initially intended for pain relief; sildenafil for hypertension and angina; and thalidomide for morning sickness; which have since been adapted for various other medical conditions [2]. Examples are relatively sparse for drugs from the field of infectious diseases. Notable instances include the repurposing of ketoconazole, an antifungal, for Cushing’s syndrome, and investigations into the use of levofloxacin and tetracyclines for Alzheimer’s disease [3]. Conversely, repurposing antimicrobials for different infectious diseases than their initial targets have proven beneficial, as seen with tetracycline for malaria [3] and linezolid for tuberculosis [4].

**Table 1:** Two distinct definitions of drug repurposing or repositioning

Definition	Comment
“They defined drug repositioning as the process of finding new uses for existing drugs, sometimes but not necessarily when they fall into the public domain and become generic drugs.” “This original definition … [was]… extended to include active substances that failed the clinical phase of their development on account of their toxicity or insufficient efficacy, as well as drugs withdrawn from the market because of safety concerns. It should not … include substances that have not yet been subjected to clinical investigation.” [42]	Only for approved drugs
“Drug repurposing … is a strategy for identifying new uses for approved or investigational drugs that are outside the scope of the original medical indication” [1]	Includes investigational drugs, requires a costly and lengthy clinical trial process, which contradicts the idea that drug repurposing is faster and less expensive

Note: Clinical investigation refers to any form of study involving human subjects that assesses the safety, tolerability, pharmacokinetics, or efficacy of a drug, which is a prerequisite for the drugs considered in our review on repurposing

Recently, a tendency for a broader definition of repurposing has surfaced (Table 1), which includes the ‘repurposing’ of not only approved drugs but also investigational drugs which are being developed for other indications [1], often including novel computational models [5, 6]. While this approach is promising to identify novel compounds, most molecules may need modifications and must still pass through the demanding clinical evaluation process. Incorporation of investigational drugs requires a costly and lengthy clinical trial process, which contradicts the idea that drug repurposing is per se faster and less expensive.

Drug repurposing initiatives targeting new antimicrobial treatments typically begin with in vitro assays or, in more advanced stages, with pre-clinical rodent models. This approach has been a common thread in both bacterial and viral research [6–9]. However, the potential for an enormous disconnect between laboratory promise and clinical efficacy is highlighted using chloroquine and ivermectin in treating SARS-CoV-2. Despite initial laboratory indications suggesting potential, both compounds ultimately demonstrated no significant efficacy [10].

Tuberculosis (TB), caused by *Mycobacterium tuberculosis* complex (MTC), remains a leading cause of death globally, with approximately 1.6 million fatalities in 2022. Furthermore, the increasing prevalence of multidrug-resistant (MDR) and extensively drug-resistant (XDR) TB poses significant treatment challenges [11, 12]. Consequently, there is an urgent need for novel anti-TB drugs. Trying to face this challenge, the past two decades have witnessed advancements such as the introduction of new anti-TB compounds (delamanid, bedaquiline) or novel treatment regimens intended to shorten treatment duration [13, 14].

Notably, the use of the existing antibiotic linezolid for TB treatment is often mentioned as a prime success story in drug repurposing [15]. Contrary to this, the use of the antibiotic levofloxacin for TB treatment is less commonly seen as 'true' repurposing; and is often simply considered an extension of the range of bacterial pathogens which are treatable with the drug. Despite these advances, the demand for new and repurposed anti-TB drugs continues unabated, addressing future issues of resistance, reducing toxic effects, identifying more effective drugs, and considering economic implications.

Compounds in the two major anti-malarial drug classes, quinolones and artemisinins, were developed and introduced exclusively for treating malaria. However, their potential for repurposing has been recognized, as several non-infectious disease indications have been well-established over time; or are currently being investigated (Table 2). Additionally, these drugs have been employed, or are under investigation, for treating various infections (Table 2).

**Table 2:** Examples of repurposing of anti-malarial drugs

Drug (class)	Repurposed use	Comment	Refs.
(Hydroxy) chloroquine	Rheumatoid arthritis (RA); Systemic Lupus Erythematosus (SLE)	Used as immune-modulating (disease -modifying antirheumatic drug)	[16–19]
	Autophagy-related pathologies	Investigated for several diseases mainly in the field of cancer and neurogenerative disorders	
	Amoebiasis	Used, in cases of liver abscess as early as the 1950s	
	HIV	Was believed to have some anti-viral effect and immune-modulating effect. Clinical trials provide little evidence for effectiveness	
	Q-Fever	Reduces treatment duration and relapses of Q fever endocarditis when combined with doxycycline	
	Viral infections	Chloroquine showed in-vitro effects against many viruses, including HIV, Ebola, SARS-CoV-1, MERS. Failed in clinical studies against COVID19	
4-aminoquinolines (mefloquine, amodiaquine, piperaquine	Schistosomiasis	In combination with artemisinins	[20]
Quinine	Leg cramps	Off-label use for the treatment of nocturnal leg cramps, although this use is controversial due to potential adverse effects	[21]
Primaquine	*Pneumocystis jiroveci* Pneumonia (PCP)	In combination with clindamycin as salvage treatment of PCP	[22]
Artemisinins	Cancer	Studies suggest that these compounds can induce apoptosis (programmed cell death) in cancer cells	[23, 24]
	Inflammatory diseases	Investigated for RA, SLE, and others	
	Schistosomiasis	Effective against young helminths (potential for combination therapy with praziquantel), also in combination with 4-aminoquinolines (see above)	
	Viral Infections	Potential antiviral effects against a range of viruses, including Hepatitis B and C, human herpesvirus, and others (not clinically confirmed)	

Amidst the global scramble to find effective treatments against COVID-19, anti-malarials emerged prominently in research and clinical trials as repurposed drugs, albeit without proven efficacy. However, this spotlight on anti-malarial drugs reinvigorated interest in their repurposing potential, which could be pivotal in addressing other infectious diseases, notably tuberculosis (TB). Recognized for its efficiency and speed, drug repurposing is especially crucial in combatting difficult-to-treat pathogens for example those which develop resistance, including MDR and XDR-TB strains. Anti-malarials have also been the subject of research for their potential as repurposed drugs for TB [8, 24, 25]. Therefore, the objective of this review is to assess the impact of anti-malarials' repurposing for TB to determine whether such efforts have been effective, and should be pursued in the future; or rather reconsidered.

## Methods

For this review, a PubMed search conducted in December 2023 focused on studies of anti-malarial drugs demonstrating in vitro and in vivo (using animal models) effects against *Mycobacterium tuberculosis*, or clinical effects on the treatment of tuberculosis. The search terms used were ‘antimalarial drugs tuberculosis’. Additionally, each individual antimalarial drug (Additional file 1: Table S1) of the two classes of quinolones and artemisinins was searched in combination with the term ‘tuberculosis’ (Additional file 1: Table S2). Furthermore, four clinical trial databases were screened for additional studies on this topic (EU Clinical Trial Register, ClinicalTrials.gov, BASEC, and ISRCTN registry). References in all relevant articles and their respective citations were also reviewed. All articles were screened based on their title and abstract using Rayyan Systems Inc (https://www.rayyan.ai/), and included studies that provide original data on the activity of established anti-malarial drug compounds (Additional file 1: Table S1) against MTC or their use in TB treatment.

Excluded were studies on anti-malarial agents initially developed as antibiotics for other infections, such as tetracyclines and clindamycin. Also omitted were drugs from classes primarily intended for non-malarial infections, such as those affecting folate synthesis (e.g., proguanil, pyrimethamine, sulfamethoxazole, or dapsone). Studies reporting in silico work, computer models, or investigations of novel compounds based on modifications of the molecular structures of existing anti-malarial drugs were also excluded. Consequently, compounds that have not yet been subjected to clinical investigation (see Table 1) were also excluded.

Study results were summarized according to their respective main methodologies applied, which were classified as: (i) In vitro activity of anti-malarial drugs against MTC in culture or in macrophage models, providing relevant quantitative measurements; (ii) in vitro activity of anti-malarial drugs against MTC in combination with other drugs (synergistic effects) in culture or in macrophage models; (iii) pre-clincial in vivo studies (animal models); and (iv) clinical trials in humans.

The publishing journal was assessed by verfiying the H-index reported by Scimago (https://www.scimagojr.com/) on 14/01/2024 which uses the Wikipedia defintion (https://en.wikipedia.org/wiki/H-index).

## Results

A PubMed search yielded 1,187 hits, with an additional 86 identified through the screening of reference lists, ultimately culminating in 19 relevant papers (Additional file 1: Fig. S1). Upon closer examination, these papers encompass a range of study types: there are 19 instances of in vitro research reported within these papers, of which five investigate synergistic effects specifically. Additionally, there are three instances of in vivo animal research and a single ongoing Phase 1 clinical trial retrieved from ClinicalTrials.Gov (Table 3, Additional file 1: Tables S3–S6) [26–44]. These studies, predominantly published between 2006 and 2021, with a peak from 2016 to 2019, explored anti-malarial drug activity against MTC. Journal impact, assessed by Scimago H-indices, varied with most journals scoring over 100 (Additional file 1: Tables S3–S6). Notably, one 1990 study [27] on chloroquine had significant methodological limitations and high inhibitory concentrations compared to recent research (Additional file 1: Tables S3, S4). In several studies, the examination of the molecular structure of some anti-malarials yielded secondary outcomes related to anti-malarial drug activity against MTC [26, 36–39]. Other research compared anti-MTC activity of anti-malarial drugs to novel compounds [29, 30, 33, 40] or assessed activity against dormant MTC strains [28, 29, 31]. Eighteen studies focused on *in-vitro* anti-malarial activity against MTC (Additional file 1: Table S3), while five explored synergies with anti-TB drugs (Additional file 1: Table S4). Only three reported *in-vivo* effects in animal models (Additional file 1: Table S5).

**Table 3:** Summary of studies (n = 19) investigating the anti-malarial drug effects against *M. tuberculosis* complex

Area/studies (n)	Anti-malarial drugs (n)	Methods (n)*	Key results**
A) *In-vitro* growth (n = 18)	mefloquine (11), chloroquine (4), primaquine (2), tafenoquine (1), amodiaquine (1), pyronaridine (1), artemisinin (3), artesunate (2)	Strains: H37Rv (12), clinical isolates (7), Erdman (3), H37Ra (2), BCG (1), others (2) Growth: REMA (7), MABA (6), OD (2), GFP (2), solid media: (4), BACTEC (1), MGIT: (1) Dormancy: LORA (2), Wayne (1), other (1)	MIC (µM): chloroquine: 2, > 62, > 125; mefloquine: 4–12, 13, 13, 13, 21, 21, 21, 33, 33, 43, 52, (11–42), (11–21); mefloquine (dormant): 7, 7, 21; primaquine: 1, 5; tafenoquine: 10–20; amodiaquine: > 56; pyr: 5; artemisinin: 265, 265, 709, > 1063; artesunate: 195, > 1562
B) *In-vitro* synergy (n = 5)	mefloquine (2), chloroquine (2), tafenoquine (1), artemisinin (1)	Strains: H37Rv (3), H37Ra (2), Erdman (1) BCG (1), others: (2) Drugs: isoniazid (5), pyrazinamide (3), rifampicin (2), streptomycin (2) ethambutol (1), others: (2) Checkerboard – FICI (n): GFP (1), MABA (1), REMA (1) Kill kinetics (1); Macrophages (2)	chloroquine + insoniazid or pyrazinamide: 0,5–1 log CFU/ml reduction chloroquine + isoniazid or pyrazinamide: no synergistic effect (indifferent) Tafenoquine + isoniazid or moxifloxacin or rifampicin or streptomycin: no synergistic effect (indifferent), only tafenoquine + mefloquine (FICI = 0,5) FICI < 0,5: mefloquine + isoniazid, (ofloxacin-streptomycin resistance) or pyrazinamide (H37Rv); FICI = 0,5 mefloquine + insoniazide (R37Rv), mefloquine + various quinolones, n = 7), no synergistic effect observed (indifferent) with various quinolines (n = 10) FICI < 0,5: artemisinin + amikacin (BCG), artemisinin + isoniazid (H37Rv); FICI = 0,5: artemisinin + isoniazid, ethambutol (BCG), artemisinin + rifampicin (H37Rv), no synergistic effect (indifferent) (various drugs tested in combination)
C) In vivo animal model, (n = 3)	mefloquine (1), chloroquine (1), artemisinin (1), artesunate (1)	H37Rv (3) / mice, guinea pigs (1), rats (1), mice (1) Infection: aerosol (2), i.v. (n = 1) / infection (4 weeks) + Tx (48 weeks) (1), infection (1 week) + Tx (4 weeks), infection (4 weeks) + Tx 4 weeks) (2) CFU counts: lung (2), lung and spleen (1) Histopathology score: lung tissue (0–4, 0 = none)	log CFU/mL in mice: drug free = chloroquine: 10^6^, inh: 10^3^, isoniazid + chloroquine: sterilizes tissue / guinea pigs: drug free: 10^6–7^, chloroquine: 10^5^, inh: 10^3^, isoniazid + chloroquine: 100 / histology: isoniazid + chloroquine score = 1–0 / reduction of relapse rate after treatment with chloroquine + isoniazid (chloroquine: 10 mg/kg i.p.) log CFU/ml in mice: mefloquine: 1,2–1,8 log CFU/ml reduction. (mefloquine 40 mg/kg p.o.) log cfu/ml in mice: artemisinin: 5 log CFU/ml reduction, artesunate: ~ 3 log CFU/ml reduction. (artemisinin and artesunate: 3,5 mg/kg p.o.)

^*^ A) Strain Descriptions: Refer to Table S3. Four studies utilized dormancy models based on nutrient/oxygen deprivation. Methods: Solid media (colony count, one study specified ‘proportion method’), macrophages (1 × human, 2 × THP-1 cell line). B) Strain Descriptions: Refer to Table S3. Drugs: One study used amikacin, another used multiple fluoroquinolones (ciprofloxacin, gatifloxacin, levofloxacin, ofloxacin, sparfloxacin)

^**^ MIC (µM): All results displayed. Notes: a) MIC50 & MIC90 = 21 (mefloquine); b) 2 studies on mefloquine and enantiomers (*erythro*/*threo* ±), *threo* less effective, mefloquine is racemic *erythro* mefloquine; c) One MBC dor mefloquine: 4–8; d) mefloquine range for multiple clinical isolates including MDR: 11–42, 11–21; e) Three MTC results in dormant model with variable comparisons to isonaizide and rifampicin; f) artemisinin & artesunate ineffective (> 1063, > 1562) in traditional liquid/solid sensitivity tests compared to REMA. FICI Interpretation: ≤ 0.5 synergism, ≥ 4 antagonism, 0.5–4 additivity/indifference

Abbreviations: CFU: Colony-Forming Units, FICI: Fractional inhibitory concentration index, GFP: Green Fluorescent Protein, H37Ra: non-virulent strain of *M. tuberculosis*, H37Rv: virulent strain of *M. tuberculosis*, LORA: Low Oxygen Recovery Assay, MABA: Microplate Alamar Blue Assay, MBC: Minimum Bactericidal Concentration, MDR: Multidrug-Resistant, MGIT: Mycobacterial Growth Indicator Tube, MIC: Minimum Inhibitory Concentration, MTC: *Mycobacterium tuberculosis* Complex, REMA: Resazurin Reduction Microplate Assay, TB: Tuberculosis, Tx: treatment

Detailed references provided in text and supplementary tables

In vitro studies predominantly investigated quinolines, especially mefloquine, chloroquine, primaquine, tafenoquine, amodiaquine, and pyronaridine, while artemisinins and artesunate were less common (Table 3). These studies employed a variety of methodologies, notably viability detection assays like resazurin and Alamar blue. Some studies used liquid (BACTEC, MGIT) or solid media, which generally showed higher MIC values compared to the viability detection assays (Tables 3, Additional file 1: Tables S3, S4). Synergistic effects in vitro mainly involved isoniazid, pyrazinamide, and quinolones, with one study examining rifampicin and artemisinins [36] (Table 3, Additional file 1: Table S4).

The main findings are summarized in Table 4. All minimum inhibitory concentration (MIC) values were significantly higher than those for the breakpoints for anti-tuberculosis drugs (rifampicin: 1.2 µM = 1.0 µg/mL and isoniazid: 1.5 = 0.2 µg/mL) against MTC and half-maximal inhibitory concentration (IC_50_) values for anti-malarial drugs against *Plasmodium falciparum*. Notably, over 100 clinical isolates [32] showed MICs ranging from 11 – 42 µM for mefloquine. No inhibition by artemisinins was observed using MGIT or Ogawa solid media, in contrast to the resazurin-based method (Tables 3, Additional file 1: Table S3) [35]. Studies on mefloquine’s MICs in dormancy models showed similar effects to those in replicating MTC [29, 31, 40], with one study indicating equivalence to rifampicin in dormant MTC (MIC for mefloquine = rifampicin: 7 µM) [29]. Interestingly, a 1990 study using macrophage models reported chloroquine’s intracellular effect to be 50 times stronger than in culture [27], whereas other studies found more modest effects for mefloquine [31, 34] (Additional file 1: Table S3).

**Table 4:** Values for MIC, cytotoxicity and serum concentration of anti-malarial drugs compared to two major anti-tuberculosis drugs (all values in µM)^*^

Drug	MIC values against MTC	IC_50_ against *P. falciparum*	IC50% (cytotoxicicty)	Serum concentrations
Chloroquine	2, > 62, > 125	0.006–0.03	37 (17–2)	0.3–1.3 / 2.5
Mefloquine	4–12, 13, 13, 13, 21, 21, 21, 33, 33, 43, 52, (11–42), (11–21)	0.006–0.04	13 (9–8)	2.6
Pyronaridine	5	0.001–0.01	~ 10	0.13
Artemisinin	265, 265, 709, > 1063	0.01–0.02	~ 100	0.4–0.7
Rifampicin**	1.2 (= 1.0 µg/mL)	1–3	128	~ 5
Isoniazid**	1.5 (= 0.2 µg/mL)	–-	> 100	~ 44

^*^ Values for anti-malarial drugs from Table 2. ** Rifampicin and Isoniazid MIC breakpoint values (also in µg/mL) as reference

Detailed list of references from which all other values were retrieved in Table S7 (supplementary file). IC_50_: half maximal inhibitory concentration, MIC: Minimum Inhibitory Concentration, MTC: *M. tuberculosis* complex

Synergistic effects were negligible or modest for the anti-malarials chloroquine and tafenoquine [27, 28, 37] (Additional file 1: Table S3). However, the fractional inhibitory concentration index (FICI) of < 0.4 indicated synergy for mefloquine combined with pyrazinamide or isoniazid, and for artemisinin with isoniazid or amikacin [26, 36] (Additional file 1: Table S3). Other results for different drug combinatons showed a FICI of around 0.5, the threshold for synergism (Table 3, Additional file 1: Table S3).

In vivo studies demonstrated some effect of chloroquine, mefloquine, or artemisinins (Tables 3, S5). Most notably, in vivo studies in mice and guinea pigs found that combining isoniazid and chloroquine produced strong effects by various metrics [39] as specified in Tables 3 and Additional file 1: Table S5.

Regarding clinical trials, only one phase 1 trial was identified. This ongoing trial examines the tolerability of chloroquine combined with a standard quadruple TB drug regimen in healthy volunteers; results pending (Additional file 1: Table S5).

## Discussion

The concept of repurposing clinically tested or approved drugs (Table 1), due to their established safety profiles, is a compelling approach for rapidly identifying new treatments. This strategy has also been applied to anti-malarial drugs for potential use in treating tuberculosis. Research often centres on the underlying structures of anti-malarials, using them as scaffolds for new compound development. This approach, illustrated by a review on quinolones [45], typically involves clinically non-tested compounds. Yet, this approach has been questioned for possibly negating the inherent benefits that were the original goal of reusing existing medications (Table 1). Chloroquine’s possible use as tuberculosis treatment is briefly discussed in a review by Rolain et al. [46], as is mefloquine [47]. A recent review expands on various anti-malarial drugs, although rather focusing on their possible mode of actions [48]. While all these reviews paint a rather optimistic picture of repurposing anti-malarial drugs, an in-depth review of the in vitro data on MIC values, synergistic effects, and in vivo data from animal models (Table 3, Additional file 1: Tables S3-S6) provides less support for this view.

### Activity of anti-malarial drugs

Most minimum inhibitory concentration (MIC) data were obtained using colorimetric redox indicator assays (Additional file 1: Table S3), offering a rapid and simplified method for assessing drug resistance and screening new drugs [49]. However, MIC values determined through traditional liquid or solid media, which are standard in clinical microbiology, were significantly higher for mefloquine [32] and artemisinins [35]. Also, two studies which tested 113 and 228 clinical isolates, respectively [32, 41], reported higher MICs than those studies which used only a few laboratory-adapted strains (majority ≥ 21 μM mefloquine as compared to 4-12 μM, respectively) (see Tables 4, Additional file 1: S3 for details).

It is crucial to highlight that the MIC values for anti-malarial drugs are significantly higher compared to those reported for rifampicin and isoniazid, as demonstrated in Table 4. Additionally, the MIC values for these anti-malarial drugs against MTC are also markedly higher compared to their IC_50_ values against *P. falciparum* (Table 4). The serum levels of anti-malarials exceed the IC_50_ for *P. falciparum* which, interestingly, also includes rifampicin (Table 4). This explains why rifampicin has shown to have acceptable efficacy when combined with cotrimoxazole and isoniazid in clinical trials for the treatment of malaria, although parasitological cure rates were inferior [50]. However, the serum concentrations of anti-malarial drugs are 1–2 orders of magnitude lower than the MIC values for MTC. This disparity suggests that effective concentrations against MTC might only be achievable with significantly increased drug dosages.

It has been suggested that such MIC values could be achieved intracellularly [32]; however, the few studies that investigated this in macrophages report only modest effects [27, 31, 34, 42].

*In-vitro* studies show ambiguous results regarding the synergistic effects of anti-malarial and anti-TB drugs (Table 3, Additional file 1: Table S4). These effects are gauged using the fractional inhibitory concentration index (FICI) from a checkerboard assay, categorizing ≤ 0.5 as synergism, ≥ 4 as antagonism, and > 0.5—< 4 as indifferent or independent. Often, drugs considered synergistic barely meet the 0.5 FICI threshold (Table 4, Additional file 1: Table S4). Studies on dormant bacteria models (Additional file 1: Table S3) revealed similar MIC values for mefloquine and rifampicin at 7 µM [29], but this was primarily due to increased rifampicin MIC in dormant bacteria rather than its activity against active MTC. The clinical significance of these observations is unclear.

In assessing the activity of anti-malarial drugs in animal models for tuberculosis, the overall findings present a varied picture. Mefloquine and artemisinins, for example, demonstrated only moderate reductions in bacterial counts, ranging from 1.2 to 1.7 log CFU/mL and 2 to 3 log CFU/mL, respectively [33, 35]; while chloroquine use in mice and guinea pigs showed almost no impact on bacterial counts [39]. However, the addition of isoniazid to chloroquine led to considerable decreases in bacterial counts, sometimes resulting in sterilisation, along with reductions in histological lesion severity as well as relapse rates [39] (Tables 4, Additional file 1: Tabe S5).

Perhaps in line with this, the only identified clinical trial is an ongoing phase 1 trial which explores the safety of adding chloroquine to standard anti-TB medication in healthy volunteers (Additional file 1: Table S6).

### Toxicity

The elevated MIC values of anti-malarial drugs against MTC, in contrast to achievable serum levels (Tables 3, 4), raise significant concerns about toxicity at higher dosages. The selectivity index (SI), calculated as the ratio of IC_50%_ toxicity in cell assay to MIC, reveals low values for anti-malarials (single digits), suggesting a narrow therapeutic window, unlike the much higher values for rifampicin and isoniazid (based on values in Table 4). Consequently, serum concentrations approaching the MIC values for MTC could result in considerable toxicity, as evidenced by chloroquine toxicity studies in overdose, which conclude that death rates rise steeply at concentrations > 10 µmol/L [51].

It is important to note that dosages used in animal models for TB (Additional file 1: Table S5) are already at the higher end of what is recommended for malaria. Prolonged use of these drugs in TB treatment, which spans several months and involves patients with significant systemic infection, raises further safety concerns. Chloroquine and mefloquine have yielded an overall acceptable general safety profile in long-term malaria-chemoprophylactic use [52, 53], as well as chloroquine use in rheumatoid diseases [54]. However, specific long-term effects when added to other, potentially toxic TB treatment regimens must be carefully considered, as the case of prolonged use of linezolid illustrates [55].

### Other implications and consequences

Repurposing antimicrobials for non-infectious diseases rises concerns about increasing antimicrobial resistance, though conclusive data are currently lacking [3]. The use of tetracyclines for malaria or linezolid and levofloxacin for TB, may have contributed to bacterial resistance; but a significant public health impact has not been reported so far. A key consideration in repurposing anti-malarials for TB treatment is the co-endemicity of malaria and TB, particularly in sub-Saharan Africa, where they affect overlapping populations. The prolonged use of anti-malarials for many months in TB treatment could potentially increase the risk of *Plasmodium* spp. parasites developing resistance. Certainly, this is of utmost importance for artemisinins. Contrary to this, chloroquine's ongoing current primary use against *Plasmodium vivax* (although there is shift towards applying ACT in the first place also against non-falciparum human-pathogenic *Plasmodium*) reduces its perceived risk in contributing to *P. falciparum* resistance. However, continued use could still foster resistance, also affecting the effectiveness of partner drugs in artemisinin-based combination therapy, such as mefloquine, lumefantrine, and amodiaquine, due to cross-resistance (markers: *pfcrt* and *pfmdr1*) [56]. Certainly, the long-term use of chloroquine in other situations, like in treating rheumatoid arthritis, affects only a relatively small and distinct overlapping population, and is unlikely to significantly influence *Plasmodium* resistance. Intriguingly, long-term chloroquine use seems to provide some limited protective effect against TB in rheumatoid patients undergoing immunosuppressive therapy [57, 58], although it was also associated with increased incidence of non-tuberculous mycobacterial infections [58].

## Conclusions

The analysis has reviewed the possibility of repurposing anti-malarial drugs by leveraging their anti-mycobacterial properties for TB treatment. However, this potential appears to be limited by high MIC values, poor synergy, and minimal in vivo effects. Concerns about potential toxicity at effective dosages and the risk of antimicrobial resistance, especially where TB and malaria overlap, further question their repurposing. More importantly, it prompts a critical question: Is such a strategy both viable and beneficial? The co-endemicity of tuberculosis and malaria across many regions presents scenarios where co-infections are common, and using a drug to treat one condition may inadvertently influence the pathogen responsible for the other.

While the repurposing of medications like linezolid and fluoroquinolones has become integral to TB management, potentially impacting bacterial populations, the possible repercussions of such resistance appear to be manageable in public health terms, given the availability of alternative antibiotic classes and the possibly limited transmission of such bacteria. In contrast, the idea of adapting anti-malarials for TB treatment raises significant concerns due to the potential for inducing resistance in local parasite strains, thereby compromising the effectiveness of the limited arsenal of anti-malarial drugs.

Therefore, the clinical value of repurposing anti-malarials for TB is questionable, with scepticism regarding its ability to substantially alter TB treatment paradigms. In the light of these considerations, a cautious stance is advised, recommending against the systematic exploration of anti-malarials as TB treatments. The associated risks of resistance development, combined with limited chances of revolutionizing clinical practice, support this cautious approach. The pursuit of novel TB treatments should instead focus on strategies offering clear benefits and presenting a reduced risk of aggravating the global challenge of infectious diseases.

### Supplementary Information

**Additional file 1.** The additional file includes a detailed search strategy and comprehensive tables summarizing the results from all included studies.
