# Supplementary material for: Repurposing of anti-malarial drugs for the treatment of tuberculosis: realistic strategy or fanciful dead end?
Source: Malar J. 2024 May 3;23:132. doi: 10.1186/s12936-024-04967-2 (PMC11067164; doi:10.1186/s12936-024-04967-2)
Supplement: Supplementary file 1 — Additional file 1. The additional file includes a detailed search strategy and comprehensive tables summarizing the results from all included studies. [file 12936_2024_4967_MOESM1_ESM.docx]

**SUPPLEMENTARY FILE:**

**Repurposing of antimalarial drugs for the treatment of tuberculosis: realistic strategy or fanciful dead end?**

Thomas Hanscheid^1^, Claire Ruiz del Portal Luyten^2^, Sabine M. Hermans^2^, Martin P. Grobusch^2,3,4,5,6*^

^1^Instituto de Microbiologia, Faculdade de Medicina, Universidade de Lisboa, Lisbon, Portugal

^2^Center for Tropical Medicine and Travel Medicine, Department of Infectious Diseases, Division of Internal Medicine, Amsterdam UMC, Location University of Amsterdam, Amsterdam, Netherlands

^3^Institute of Tropical Medicine, German Centre for Infection Research (DZIF), University of Tübingen, Tübingen, Germany

^4^Centre de Recherches Médicales en Lambaréné (CERMEL), Lambaréné, Gabon

^5^Masanga Medical Research Unit (MMRU), Masanga, Sierra Leone

^6^Institute of Infectious Diseases and Molecular Medicine (IDM), University of Cape Town, Cape Town, South Africa

**Content:**

**Item Page**

Table S1 - PubMed search terms and translations 2

Table S2 - List of antimalarial drugs included in the review process 3

Table S3 - Summary of *in vitro* results of antimalarial drug effects against

*Mycobacterium tuberculosis* complex (MTC) 4

Table S4 - Summary of *in vitro* results of antimalarial drug effects

(in synergy) against *Mycobacterium tuberculosis* complex (MTC) 8

Table S5 - Summary of preclinical *in vivo* studies (animal models)

investigating efficacy of antimalarial drugs on *M. tuberculosis* complex (MTC) 10

Table S6 - Summary of clinical studies investigating antimalarial drugs

for TB treatment 11

References for Tables S3-S6 11

Table S7 - References (Sources) for information and values in table 3 of

main document. 13

Figure S1 - Flow Chart of the Literature Review Process 15

**Table S1 PubMed search terms and translations**

| **Search** | **Query (Search)** | **Results** |
| --- | --- | --- |
| **#14*** | **#1 OR #2 OR #3 OR #4 OR #5 OR #6 OR #7 OR #8 OR #9 OR #10 OR #11 OR #12 OR #13** | **1187** |
| #13 | **(artemisinins OR artemether OR artesunate OR artenimol OR dihydroartemisinin) tuberculosis** | 123 |
| #12 | **atovaquone tuberculosis** | 15 |
| #11 | **tafenoquine tuberculosis** | 2 |
| #10 | **primaquine tuberculosis** | 23 |
| #9 | **pyronaridine tuberculosis** | 1 |
| #8 | **piperaquine tuberculosis** | 5 |
| #7 | **mefloquine tuberculosis** | 36 |
| #6 | **lumefantrine tuberculosis** | 26 |
| #5 | **halofantrine tuberculosis** | 0 |
| #4 | **halofantrine tuberculosis - Schema: all** | 0 |
| #3 | **(chloroquine OR hydroxychloroquine) tuberculosis** | 209 |
| #2 | **quinine tuberculosis** | 44 |
| #1 | **antimalarial drugs tuberculosis** | 1037 |

| **Translations: (#14)**  **antimalarial drugs:** "antimalarials"[Pharmacological Action] OR "antimalarials"[MeSH Terms] OR "antimalarials"[All Fields] OR ("antimalarial"[All Fields] AND "drugs"[All Fields]) OR "antimalarial drugs"[All Fields]  **tuberculosis:** "tuberculosi"[All Fields] OR "tuberculosis"[MeSH Terms] OR "tuberculosis"[All Fields] OR "tuberculoses"[All Fields] OR "tuberculosis's"[All Fields]  **quinine:** "quinine"[MeSH Terms] OR "quinine"[All Fields] OR "quinines"[All Fields]  **tuberculosis:** "tuberculosi"[All Fields] OR "tuberculosis"[MeSH Terms] OR "tuberculosis"[All Fields] OR "tuberculoses"[All Fields] OR "tuberculosis's"[All Fields]  **chloroquine:** "chloroquin"[All Fields] OR "chloroquine"[MeSH Terms] OR "chloroquine"[All Fields] OR "chloroquine's"[All Fields] OR "chloroquines"[All Fields]  **hydroxychloroquine:** "hydroxychloroquine"[MeSH Terms] OR "hydroxychloroquine"[All Fields]  **tuberculosis:** "tuberculosi"[All Fields] OR "tuberculosis"[MeSH Terms] OR "tuberculosis"[All Fields] OR "tuberculoses"[All Fields] OR "tuberculosis's"[All Fields]  **lumefantrine:** "lumefantrine"[MeSH Terms] OR "lumefantrine"[All Fields]  **tuberculosis:** "tuberculosi"[All Fields] OR "tuberculosis"[MeSH Terms] OR "tuberculosis"[All Fields] OR "tuberculoses"[All Fields] OR "tuberculosis's"[All Fields]  **mefloquine:** "mefloquine"[MeSH Terms] OR "mefloquine"[All Fields] OR "mefloquin"[All Fields]  **tuberculosis:** "tuberculosi"[All Fields] OR "tuberculosis"[MeSH Terms] OR "tuberculosis"[All Fields] OR "tuberculoses"[All Fields] OR "tuberculosis's"[All Fields]  **piperaquine:** "piperaquine"[Supplementary Concept] OR "piperaquine"[All Fields]  **tuberculosis:** "tuberculosi"[All Fields] OR "tuberculosis"[MeSH Terms] OR "tuberculosis"[All Fields] OR "tuberculoses"[All Fields] OR "tuberculosis's"[All Fields]  **pyronaridine:** "pyronaridine"[Supplementary Concept] OR "pyronaridine"[All Fields]  **tuberculosis:** "tuberculosi"[All Fields] OR "tuberculosis"[MeSH Terms] OR "tuberculosis"[All Fields] OR "tuberculoses"[All Fields] OR "tuberculosis's"[All Fields]  **primaquine:** "primaquine"[MeSH Terms] OR "primaquine"[All Fields] OR "primaquine's"[All Fields]  **tuberculosis:** "tuberculosi"[All Fields] OR "tuberculosis"[MeSH Terms] OR "tuberculosis"[All Fields] OR "tuberculoses"[All Fields] OR "tuberculosis's"[All Fields]  **tafenoquine:** "tafenoquine"[Supplementary Concept] OR "tafenoquine"[All Fields]  **tuberculosis:** "tuberculosi"[All Fields] OR "tuberculosis"[MeSH Terms] OR "tuberculosis"[All Fields] OR "tuberculoses"[All Fields] OR "tuberculosis's"[All Fields]  **atovaquone:** "atovaquone"[MeSH Terms] OR "atovaquone"[All Fields]  **tuberculosis:** "tuberculosi"[All Fields] OR "tuberculosis"[MeSH Terms] OR "tuberculosis"[All Fields] OR "tuberculoses"[All Fields] OR "tuberculosis's"[All Fields]  **artemisinins:** "artemisinin"[Supplementary Concept] OR "artemisinin"[All Fields] OR "artemisinine"[All Fields] OR "artemisinins"[MeSH Terms] OR "artemisinins"[All Fields] OR "artemisinin's"[All Fields]  **artemether:** "artemether"[MeSH Terms] OR "artemether"[All Fields]  **artesunate:** "artesunate"[MeSH Terms] OR "artesunate"[All Fields] OR "artesunic"[All Fields]  **artenimol:** "artenimol"[Supplementary Concept] OR "artenimol"[All Fields]  **dihydroartemisinin:** "artenimol"[Supplementary Concept] OR "artenimol"[All Fields] OR "dihydroartemisinin"[All Fields] OR "dihydroartemisinine"[All Fields]  **tuberculosis:** "tuberculosi"[All Fields] OR "tuberculosis"[MeSH Terms] OR "tuberculosis"[All Fields] OR "tuberculoses"[All Fields] OR "tuberculosis's"[All Fields] |
| --- |

*Results produced by “Search #14” (n=1187) were used for this review.

**Table S2 List of antimalarial drugs included in the review process**

| **Antimalarial drugs** | **Drug class** |
| --- | --- |
| Artemisinin and derivatives  (Artemether, Artesunate, Artenimol) | sesquiterpene lactones |
| Atovaquone | hydroxynaphthoquinone |
| Chloroquine /Hydroxychloroquine | 4-aminoquinoline |
| Halofantrine | phenanthrene methanol (arylamino alcohol moiety) |
| Lumefantrine | phenanthrene methanol (arylamino alcohol moiety) |
| Mefloquine | fluorinated 4-quinoline methanol (arylamino alcohol moiety) |
| Piperaquine | bisquinoline |
| Pyronaridine | benzo-naphthyridine derivative |
| Primaquine | 8-aminoquinoline |
| Quinine | naturally occurring quinoline alkaloid (arylamino alcohol moiety) |
| Tafenoquine | 8-aminoquinoline |

**Table S3 Summary of *in vitro* results of antimalarial drug effects against *Mycobacterium tuberculosis* complex (MTC)**

| **Drug** | **Mtb-strain** | **Methods*** | **Key results**** | **Remarks** | **Study / Year / journal rating (h-index)****** |
| --- | --- | --- | --- | --- | --- |
| **CQ** | Erdman | - 7H9 broth optical density - macrophage: reduction in log CFU (M) | **MIC:** none - no complete growth inhibition.  **(M) CFU/mL:** ~4 log reduction (broth) at 12.000-25.000 µM CQ | - CQ values several orders of magnitude higher than later studies.  - 1563 µM CQ in broth equivalent to 31 µM CQ in human macrophage.  - monocytes from 2 volunteers with discrepant results. | Crowle (1), 1990, H-index: 280 |
| **CQ** | H37Rv (GFP expressing) | GFP expression - checkerboard (MIC) | **MIC:**  >125 µM | No GFP measurable growth reduction at highest concentration as compared to lowest. | Matt et al. (2), 2017 (H-index:147) |
| **MQ** | - H37Rv  - 2 resistant isolates | MABA (MIC) | **MIC:**  33 µM | MIC from synergistic checkerboard study. | Dos Santos et al. (3), 2021 (H-index: 75) |
| **MQ** | H37Rv | Colony count on solid media (MIC) | **MIC:**  43 µM 16 ug/mL | From a study on proteomic analysis of combination treatments. | Danelishvili et al. (4), 2017 (H-index: 280) |
| **MQ** | - H37Rv  -228 clinical MTC Isolates | - REMA (MIC) | **MIC50:**  21 µM  **MIC90:**  21 µM | Same MIC results for all Isolates and H37Rv. | Cavanaugh (5), 2017, (H-index: 211) |
| **MQ** | H_37_Rv | - MABA and GFP for MIC, CFU count for MBC (replicating bacteria)  - LORA assay (dormant bacteria) | **MIC (**+/- *erythro*-MQ)**:**  ~4-12 µM (replicating),  7 µM (dormant)  **MBC:**  4-8 µM | - Focused on MQ +/- *threo* and *erythro* enantiomers and new derivatives; *threo* enantiomers less active, MQ is racemic *erythro*-MQ - Comparison: to RIF (MIC: 0.04-0.08 µM, MBC: 2 µM in replicating, MIC: 7 µM in dormant). - Focused on non-replicating Mtb showing MQ = RIF. - SI-index: MQ: 2-5 as compared to RIF (~1200-2400) indicating narrow therapeutic window (SI-index). | Jayaprakash (6), 2006 (H-index 114) |
| **MQ** | - H_37_Rv, - 1 MDR strain | MABA (MIC) | **MIC** (MQ):  33 µM (H37a and MDR) | 17 derivatives synthezised and tested with up to 3x lower MIC. | Gonçalves (7), 2012 (H-index 179) |
| **MQ** | - Erdman,  - H_37_Rv,  - 2 MDR strains | - BACTEC (MIC), also hyperosmolar /acidic (MIC)  - Wayne system (dormant bacteria)  - macrophage: reduction in log CFU/mL | **MIC** (+ / - *erythro* MQ)**:**  21 µM (replicating)  21 µM (dormant)  **(M) CFU/mL:** 2-log reduction 4 µg/mL | - MQ and +/- *erythro* enantiomers similar results; +/- *threo* enantiomers less active. MQ is racemic *erythro*-MQ - MIC in one MDR: 16-32 µg/mL.  - Similar MIC values in hyperosmolar and acidic conditions. | Bermudez (8), 2014 (No H-index) |
| **MQ** | Local MTC Isolates: - 92 sensitive  - 21 MDR | - 7H10 solid media proportion method: MIC (dilution >99% inhibtion) | **MIC** (MQ):  MDR: 10,5 µM (n=4), 21 µM (n=17)  Sensitive: 10,5 µM (n=10), 21 µM (n=72), 42 µM (n=10) | No difference between MDR and sensitive strains given (we calculate a P=0.46) | Krieger (9), 2015, (H-index 269) |
| **MQ** | - H_37_Rv,  - 3 sensitive and 11ofloxacin resistant Isolates | - MABA, REMA (MIC) | **MIC** (MQ):  H37Rv: 13 µM  Clinical Isolates: 10,5 µM (n=5), 21 µM (n=9) | No results given for MQ in macrophage model which was used in study. REMA result part of synergy study. | Rodrigues (10), 2016 (H-index: 147) |
| **MQ** | H_37_Rv | - MABA (MIC) (replicating bacteria) - LORA (MIC) (dormant bacteria) | **MIC** (MQ):  - replicating: 13 µM  - dormant: 7 µM | - MQ at 40-80 mg/kg po p/d for 4 days shows a 5-10% reduction in body weight.  - MQ Cytotoxicity in VERO cells: IC_50_: 11 µM | Mao et al. (11), 2007, (H-index: 141) |
| **CQ, MQ, PQ, TQ** | - H_37_Ra  - H_37_Rv,  - 3 MDR strains | - REMA (MIC) (replicating)  - CFU reduction in nutrient starvation (dormant)  - kill kinetics (KK) | **MIC**:  CQ: 2 µM; PQ: 1,4 µM (H37Ra) MQ: 21 µM TQ: 10-20 µM (all strains)  **(KK) CFU/mL:** ~3 log reduction (20, 40, 80 µM) TQ at day 14. | - Killing kinetics show ~4 log CFU/mL growth for RIF and INH although 5x and 300x MIC concentration used.***  - Note that a ~0.5-1 log CFU increase from day 10 to 14 occurred (regrowth?).  - TQ, MQ similar inhibtion as RIF or INH (log-phase), slightly better inhibition in dormant bacteria. | Sidrônio (12), 2021, (H-index: 94) |
| **CQ, MQ, AMO, PYR** | H37Rv | - REMA  - solid media colony count  - macrophage assay (inhibiton of growth) | **MIC**:  MQ: 52 µM, CQ: >62 µM, AMO: >56 µM, PYR: 5 µM.  14 µM (PYR) in macrophage assay | - Provide MIC for PYR in macrophage assay (THP 1 cell line) as value for 100% growth inhibition. PYR had no effect up to 3.1 µg/mL in murine macrophage. | Mori (13**),** 2018, (H-index: 94) |
| **PQ** | H_37_Rv | - MABA (MIC)  - macrophage assay (inhibiton of growth) | **MIC** (PQ):  5 µM  - Macrophage assay: inhibition growth: 5 µM | - No quantification (CFU-reduction) in macropahge assay. - No list of the 1514 compounds which were tested give, but possible that other antimalarial drugs were tested and deemed ineffective (cut-off: 5 µM). | Lougheed (14), 2009, (H-index: 94) |
| **ARM, ART** | H_37_Rv | REMA, MGIT, Ogawa medium for groth detection (MIC) | **MIC:**  ARM 265 µM; ART 195 µM (REMA at day 5):  ARM 1.063 µM, ART >1.562 µM (MGIT and Ogawa (day 21) | Note: standard growth based methods (MGIT; Ogawa) detect no relevant inhibition after 21 days. | Choi (15), 2017, (no H-index) |
| **ARM** | *- M. bovis* BCG,  - H_37_Ra | - REMA (MIC)  - kill kinetics (KK) | **MIC:**  709 µM (REMA with BCG)  **(KK) CFU/mL:** < 1 log reduction at day 5 | Mainly investigation synergistic effects with Anti-TB drugs. | Patel (16), 2019, (H-index: 94) |
| **ARM** | - MTC mc26230  - Erdman | - REMA (MIC) | **MIC:**  ARM: 265 µM, ARM in *A. annua*: 138 µM | Two plant extract used from *A annua* and *A. afra* – MIC given concentration of ARM present in plant extract dilution. MIC for *A. afra* (ARM <0.37) appears disordant with text (mistake?).*** | Martini (17), 2020, (H-index: 219) |
| **ART** | - MTC CDC1551 (GFP)  - Erdman | - optical density measurement for growth | **EC_50_**: >80 µM | Studied DosRST regulon: ARM inhibits MTC CDC1551 DosRST GFP fluoresene at MIC: 12 80 µM. Little information on optical growth assessment. | Zheng (18) 2016, (H-index: 246) |

* Few studies used standard sensitivity tests, used in clinical microbiology laboratories to determine clinically useful MICs. Most used viability assays (MABA and REMA) which have been proven useful for rapid detection of resistance or for screening of drug effects, but clinical relevance of MIC determination is questionbale.

** For better comaprison, all MIC/MBC results have been provided as µM (ug/mL was transformed using standard calculation: Concentration in µM = Concentration in µg/mL×10^3^​ / Molecular Weight of drug in g/mol.

*** Respective study appears to contain a msitake – authors were contacted but have not answered to clarify the doubt.

**** Journal ranking indicator from Scimago Journal & Country Rank (https://www.scimagojr.com/)

Abbreviations: AMO: Amodiaquine, ARM: Artemisinin, ART: Artesunate, CFU: Colony-Forming Units, CQ: Chloroquine, EC_50:_ concentration for 50% maximal effect, GFP: Green Fluorescent Protein, H37Ra: non-virulent strain of *M. tuberculosis*, H37Rv: virulent strain of *M. tuberculosis*, LORA: Low Oxygen Recovery Assay, MABA: Microplate Alamar Blue Assay, MBC: Minimum Bactericidal Concentration, MDR: Multidrug-Resistant, MGIT: Mycobacterial Growth Indicator Tube, MIC: Minimum Inhibitory Concentration, MTC: *Mycobacterium tuberculosis* Complex, MQ: Mefloquine, PQ: Primaquine, PYR: Pyronaridine, REMA: Resazurin Reduction Microplate Assay, TB: Tuberculosis, TQ: Tafenoquine.

**Table S4 Summary of *in vitro* results of antimalarial drug effects (in synergy) against *Mycobacterium tuberculosis* complex (MTC)**

| **Antimalarial Drug** | **Other drugs** | **MTC-strain** | **Methods** | **Key results** | **Remarks** | **Study / Year** |
| --- | --- | --- | --- | --- | --- | --- |
| **CQ** | INH, PZA, SM | Erdman | Interaction studies with other drugs in human macrophages | - CQ (31 µM ) reduced effective SM concentration from 8,6 µM to 1,7 µM.  - CQ (31 µM) added to INH (0,3 µM) and PZA (325 µM) inceased CFU/mL reduction by ~0,5 to1 log. | Only monocyte from one human volunteer for SM and another for INH/PZA. (reproducibility?) | Crowle (1), 1990, (H-index: 280) |
| **CQ** | INH, PZA | H_37_Rv #1424 (pOLYG-Pr-GFP vector) | - Checkerboard, GFP fluoresence  - THP-1 macrophages, MOI: 1, CFU reduction after lysis | - No observable effect (indifferent) for INH and PZA at all tested CQ concentrations up to 125 µM. | In monocytes, combination (PZA + CQ) reduced CFUs more than PZA alone. | Matt et al. (2), 2017 (H-index:147) |
| **MQ** | INH, PZA, MXF, GTX, CPX, LVX, OFX, SPX | - H_37_Rv  - 2 resistant strains (see comments) | Checkerboard, MABA  MIC, FICI | **FICI (**MQ 33 µM + drug)**:**  - H37Rv: INH (0,5); PZA (0,3); MXF (0,6); GTX (1,0); CPX (1,0); LVX (1,0), OFX (0,5); SPX (1,0)  - T3609: INH (0,03); PZA (-);MXF (0,5); GTX (0,5) CPX (0,9); LVX (1,2); OFX (0,7); SPX (0,5)  - T113:INH (0,7); PZA (-);MXF (1,3); GTX (1,3); CPX (0,5); LVX (0,5); OFX (0,7) SPX (1,0) | - consult original article for other drug conentrations.  - Resistance: T3609 (OFX, SM), T113 (OFX, INH, RIF, ETB). - All but two (underlined) FITC considered synergestic: borderline value only (0.5). FICI ≤0.5 synergism; FICI ≥4 antagonism;  FICI 0,5 - 4 additivity (or indifferent or independent action) | Dos Santos et al. (3), 2021 (H-index: 75) |
| **TQ, (MQ)** | INH, RIF, ETB, SM, MXF | - H_37_Ra;  - H_37_Rv | - Checkerboard, REMA  MIC, FICI | **FICI** (TQ 10 µM) - TQ with all drugs (1 - 2): indifferent action.  - only TQ + MQ: (0,5) synergistic action | - MQ only used in combination with TQ.  - FICI considered synergestic: borderline value only (0.5). | Sidrônio (12), 2021, (H-index: 94) |
| **ARM** | INH, RIF, AMK, ETB, MXF | - H_37_Ra  - MTB (BCG) | Checkerboard, REMA,  CFU-count, MIC, FICI, kill kinetics | **FICI** (ARM 177 µM), BCG: INH (0,5), ETB (0,5), AMK (0,38), MXF (0,75)  **FICI** (ARM 88,5 177 µM for ETB, MXF; 44,25 177 µM for RIF, INH AMK) H37Ra:  RIF (0,5), INH (0,375), ETB (0,75), AMK (0,75), MXF (0,74) | - ARM MICs high, withRIF the ARM MIC reduces to 25 μg/mL; MIC of rifampicin reduced by 4-fold.  - ARM + RIF seems to kill BCG faster. | Patel (16), 2019, (H-index: 94) |

Abbreviations:AMK, amikacin; ARM, artemisinin; CFU, colony-forming units; CFX, ciprofloxacin; CLA, clarithromycin; CQ, chloroquine; ETB, ethambutol; FICI, Fractional inhibitory concentration index; GTX, gatifloxacin; INH, isoniazid; LVX, levofloxacin; MABA, microplate Alamar blue assay; MIC, minimum inhibitory concentration; MTC, *M. tuberculosis* complex; MQ, mefloquine; MXF, moxifloxacin; OFX, ofloxacin; PZA, pyrazinamide; RIF, rifampicin; REMA, resazurin reduction microplate assay; TB, tuberculosis

**Table S5 Summary of preclinical *in vivo* studies (animal models) investigating efficacy of antimalarial drugs on *M. tuberculosis* complex (MTC)**

| **Drug** | **Other drugs** | **MTC-strain** | **Animal** | **Methods** | **Key results** | **Remarks** | **Study** |
| --- | --- | --- | --- | --- | --- | --- | --- |
| **CQ** | INH, RIF | H_37_Rv | - BALB/c Mice (F)  - Guinea pigs (Hartley) | - CQ: 10 mg/kg i.p. (mice), 5mg/kg i.p. (guinea pigs); - Aerosol route infection (for 4 weeks) - CQ alone and in combination with INH or RIF (for 8 weeks)  - CFU counts in lung tissue. - histopathology scored lung damage (0-4, 0 = no damage) | - Mice (8 weeks) bacterial load: drug-free: 10^6^, INH: 10^3^, CQ: 10^6^, INH + CQ: sterile - Guinea pigs: drug-free: 10^6-7^, INH: 10^3^, CQ: 10^5^, INH + CQ: 100 - Mice (8 weeks) histopathology scores: drug-free: 4, INH: 2, CQ: 3, INH + CQ: 0-1  - Relapse in mice INH: 5 of 5, INH + CQ: 3 of 5 | - dosages: INH (25mg/kg), RIF: (10mg/kg).  - Study on phagolysosomes: - counteracting low pH by CQ administration improves INH effect on MTC. - RIF + INH showed ~1 log larger reduction compared to RIF - relapse after treatment (20 weeks post infection, dexamethosone given). | Mishra et al. (19), 2019, (H-index: 265) |
| **MQ** | - | H_37_Rv | Swiss Mice (M) | - 40mg/kg oral gavage  - intravenous, retroorbital infection (1 week)  - CFU counts in lung/spleen tissue ( after 4 weeks treatment). | Bacterial load reductions:  - lung: 1,78 log CFU/mL reduction  - spleen: 1,23 log CFU/mL reduction | - Studied novel MQ derivative (1E) which showed comparable activity to MQ. | Rodrigues (10), 2016 (H-index: 147) |
| **ARM, ART** | - | H_37_Rv | Rats (F) (Sprague Dawley) | - ARM or ART: 3,5mg/kg oral  - intranasal infection (4 weeks)  - CFU counts in lung tissue (after 4 weeks treatment). | - at 4 weeks infection: log 8 CFU/ mL  - at 8 weeks (4 weeks treatment): ARM: log 5 CFU/mL ART: ~log 3 CFU/mL (INH: log 1 CFU/mL) | - Dosage of INH: 5mg/kg.  - 1,75mg/kG and 3,5mg/kg used in toxicity study: no effects observed.  - at 8 weeks no results for drug free control mice. | Choi (15), 2017, (no H-index) |

Abbreviations: ARM: artemisinin; ART: artesunate; CFU: colony-forming units, CQ: chloroquine, F: female, INH: isoniazid, M: male, MTC:, *M. tuberculosis* complex; MQ: mefloquine, RIF: rifampicin; TB, tuberculosis, H37Rv: virulent *M. tuberculosis* complex strain.

**Table S6 Summary of clinical studies investigating antimalarial drugs for TB treatment**

| Study | Participants | Antimalarial | Other drugs | Study type | Duration study | Key results | Remarks |
| --- | --- | --- | --- | --- | --- | --- | --- |
| **Kalin M et al.** | 16 healthy volunteers (18-50 years) | CQ (100-300 mg/day) | Rimstar ® (RIF, INH, PZA ETB  (standard dose) | Phase 1 Clinical trial | 14 days of treatment + 8 months follow-up visits | n/a | No results available because the study is still recuiting |

Data retrieved from ClinicalTrials.gov (accessed 15/01/2024) (https://clinicaltrials.gov/study/NCT05443178),

Abbreviations: CQ: chloroquine, RIF: rifampicin, INH: isoniazid, PZA: pyrazinamide, ETB: ethambutol

**References for Tables S3-S6**

1. Crowle AJ, May MH. Inhibition of tubercle bacilli in cultured human macrophages by chloroquine used alone and in combination with streptomycin, isoniazid, pyrazinamide, and two metabolites of vitamin D3. Antimicrob Agents Chemother. 1990;34:2217–22. doi: 10.1128/AAC.34.11.2217.

2. Matt U, Selchow P, Dal Molin M, Strommer S, Sharif O, Schilcher K, et al. Chloroquine enhances the antimycobacterial activity of isoniazid and pyrazinamide by reversing inflammation-induced macrophage efflux. Int J Antimicrob Agents. 2017;50:55-62. doi: 10.1016/j.ijantimicag.2017.02.022.

3. dos Santos MC, Scaini JLR, Lopes MVC, Rodrigues BG, Silva NO, Borges CRL, et al. Mefloquine synergism with anti-tuberculosis drugs and correlation to membrane effects: Biologic, spectroscopic and molecular dynamics simulations studies. Bioorg Chem. 2021;110:104786. doi: 10.1016/j.bioorg.2021.104786.

4. Danelishvili L, Shulzhenko N, Chinison JJJ, Babrak L, Hu J, Morgun A, et al. Mycobacterium tuberculosis proteome response to antituberculosis compounds reveals metabolic “escape” pathways that prolong bacterial survival. Antimicrob Agents Chemother. 2017;61:e00430-17. doi: 10.1128/AAC.00430-17.

5. Cavanaugh JS, Jou R, Wu MH, Dalton T, Kurbatova E, Ershova J, et al. Susceptibilities of MDR Mycobacterium tuberculosis isolates to unconventional drugs compared with their reported pharmacokinetic/pharmacodynamic parameters. J Antimicrob Chemother. 2017;72:1678-1687. doi: 10.1093/jac/dkx022.

6. Jayaprakash S, Iso Y, Wan B, Franzblau SG, Kozikowski AP. Design, synthesis, and SAR studies of mefloquine-based ligands as potential antituberculosis agents. ChemMedChem. 2006;1:593–7. doi: 10.1002/cmdc.200600010.

7. Goncalves RSB, Kaiser CR, Loureno MCS, Bezerra FAFM, De Souza MVN, Wardell JL, et al. Mefloquine-oxazolidine derivatives, derived from mefloquine and arenecarbaldehydes: In vitro activity including against the multidrug-resistant tuberculosis strain T113. Bioorganic Med Chem. 2012;20:243–8. doi: 10.1016/j.bmc.2011.11.006.

8. Bermudez LE, Meek L. Mefloquine and Its Enantiomers Are Active against Mycobacterium tuberculosis In Vitro and in Macrophages. Tuberc Res Treat. 2014;2014:530815. doi: 10.1155/2014/530815.

9. Krieger D, Vesenbeckh S, Schönfeld N, Bettermann G, Bauer TT, Rüssmann H, et al. Mefloquine as a potential drug against multidrug-resistant tuberculosis. Eur Respir J. 2015;46:1503-5. doi: 10.1183/13993003.00321-2015.

10. Rodrigues-Junior VS, Villela AD, Gonçalves RSB, Abbadi BL, Trindade RV, López-Gavín A, et al. Mefloquine and its oxazolidine derivative compound are active against drug-resistant Mycobacterium tuberculosis strains and in a murine model of tuberculosis infection. Int J Antimicrob Agents. 2016;48:203–7. http://dx.doi.org/10.1016/j.ijantimicag.2016.04.029

11. Mao J, Wang Y, Wan B, Kozikowski AP, Franzblau SG. Design, synthesis, and pharmacological evaluation of mefloquine-based ligands as novel antituberculosis agents. ChemMedChem. 2007;2:1624-30. doi: 10.1002/cmdc.200700112.

12. Sidrônio MGS, Castelo Branco APOT, Abbadi BL, Macchi F, Silveira MD, Lock G de A, et al. Effects of tafenoquine against active, dormant and resistant Mycobacterium tuberculosis. Tuberculosis (Edinb). 2021;128:102089. doi: 10.1016/j.tube.2021.102089.

13. Mori G, Orena BS, Franch C, Mitchenall LA, Godbole AA, Rodrigues L, et al. The EU approved antimalarial pyronaridine shows antitubercular activity and synergy with rifampicin, targeting RNA polymerase. Tuberculosis (Edinb). 2018;112:98-109. doi: 10.1016/j.tube.2018.08.004.

14. Lougheed KEA, Taylor DL, Osborne SA, Bryans JS, Buxton RS. New anti-tuberculosis agents amongst known drugs. Tuberculosis (Edinb). 2009;89:364-70. doi: 10.1016/j.tube.2009.07.002.

15. Choi WH. Novel pharmacological activity of artesunate and artemisinin: Their potential as anti-tubercular agents. J Clin Med. 201710;6:30. doi: 10.3390/jcm6030030.

16. Patel YS, Mistry N, Mehra S. Repurposing artemisinin as an anti-mycobacterial agent in synergy with rifampicin. Tuberculosis (Edinb). 2019;115:146-153. doi: 10.1016/j.tube.2019.03.004.

17. Martini MC, Zhang T, Williams JT, Abramovitch RB, Weathers PJ, Shell SS. Artemisia annua and Artemisia afra extracts exhibit strong bactericidal activity against Mycobacterium tuberculosis. J Ethnopharmacol. 2020;262:113191. doi: 10.1016/j.jep.2020.113191.

18. Zheng H, Colvin CJ, Johnson BK, Kirchhoff PD, Wilson M, Jorgensen-Muga K, et al. Inhibitors of Mycobacterium tuberculosis DosRST signaling and persistence. Nat Chem Biol. 2017;13:218-225. doi: 10.1038/nchembio.2259.

19. Mishra R, Kohli S, Malhotra N, Bandyopadhyay P, Mehta M, Munshi MH, et al. Targeting redox heterogeneity to counteract drug tolerance in replicating Mycobacterium tuberculosis. Sci Transl Med. 2019;11(518):eaaw6635. doi: 10.1126/scitranslmed.aaw6635.

**Table S7 References (Sources) for information and values in table 3 of main document.**

**Values for MIC, cytotoxicity and serum concentration (all values in µM) (table 3 in main doument)**

| **Drug** | **MIC values against MTC*** | **IC_50_ against *P. falciparum* **** | **IC50% (toxicicty)***** | **Serum concentrations****** |
| --- | --- | --- | --- | --- |
| CQ | 2, >62, >125 | 0.006-0.03 | 37 (17-2) | 0.3-1.3 / 2.5 |
| MQ | 4-12, 13, 13, 13, 21, 21, 21, 33, 33, 43, 52, (11-42), (11-21) | 0.006-0.04 | 13 (9-8) | 2.6 |
| PYR | 5 | 0.001-0.01 | ~10 | 0.13 |
| ARM | 265, 265, 709, >1063 | 0.01-0.02 | ~100 | 0.4-0.7 |
| RIF***** | 1.2 (= 1.0 µg/mL) | 1-3 | 128 | ~5 |
| INH***** | 1.5 (= 0.2 µg/mL) | --- | >100 | ~44 |

* Values for antimalarial drugs from Table 2. Values for MTC from reference (1): breakpoint MIC, and (2): MTC strains from Romania.

** All values from reference (3), except (4) for PYR and (Pradines) for RIF.

*** Values for CQ (median, (25^th^-75^th^ percentile), n=32 different cell lines) reference (5), values for MQ (median, 25^th^-75^th^ percentile, n=10 different cell lines) reference (6), values for ARM reference (7), value for PYR from reference (4), value for RIF from reference (8), value for INH from reference (9).

**** Examples of achievable serum concentrations. CQ: treatment for *vivax* (10) and treatment for rheumatic disease (11); MQ: value form reference (12), PYR: C-Max values from reference (4), ARM: C-Max from reference (13), RIF and INH estimated from reference (14).

***** breakpoint values given in µM and µg/mL for reference.

ARM: Artemisinin, CQ: Chloroquine, IC_50_: half maximal inhibitory concentration, INH: Isoniazid, MIC: Minimum Inhibitory Concentration, MQ: Mefloquine, MTC: *M. tuberculosis* complex, PYR: Pyronaridine, RIF: Rifampicin.

| 1. Gumbo T. New susceptibility breakpoints for first-line antituberculosis drugs based on antimicrobial pharmacokinetic/pharmacodynamic science and population pharmacokinetic variability. Antimicrob Agents Chemother. 2010 Apr;54(4):1484-91. doi: 10.1128/AAC.01474-09. Epub 2010 Jan 19. PMID: 20086150; PMCID: PMC2849358. |
| --- |
| 1. Ruesen C, Riza AL, Florescu A, Chaidir L, Editoiu C, Aalders N, Nicolosu D, Grecu V, Ioana M, van Crevel R, van Ingen J. Linking minimum inhibitory concentrations to whole genome sequence-predicted drug resistance in Mycobacterium tuberculosis strains from Romania. Sci Rep. 2018 Jun 26;8(1):9676. doi: 10.1038/s41598-018-27962-5. PMID: 29946139; PMCID: PMC6018741. |
| 1. Rebelo M, Sousa C, Shapiro HM, Mota MM, Grobusch MP, Hänscheid T. A novel flow cytometric hemozoin detection assay for real-time sensitivity testing of Plasmodium falciparum. PLoS One. 2013 Apr 24;8(4):e61606. doi: 10.1371/journal.pone.0061606. PMID: 23637865; PMCID: PMC3634823. |
| 1. Croft SL, Duparc S, Arbe-Barnes SJ, Craft JC, Shin CS, Fleckenstein L, Borghini-Fuhrer I, Rim HJ. Review of pyronaridine anti-malarial properties and product characteristics. Malar J. 2012 Aug 9;11:270. doi: 10.1186/1475-2875-11-270. PMID: 22877082; PMCID: PMC3483207. |
| 1. Yang J, Guo Z, Liu X, Liu Q, Wu M, Yao X, Liu Y, Cui C, Li H, Song C, Liu D, Xue L. Cytotoxicity Evaluation of Chloroquine and Hydroxychloroquine in Multiple Cell Lines and Tissues by Dynamic Imaging System and Physiologically Based Pharmacokinetic Model. Front Pharmacol. 2020 Nov 20;11:574720. doi: 10.3389/fphar.2020.574720. PMID: 33658924; PMCID: PMC7919379. |
| 1. Paivandy A, Calounova G, Zarnegar B, Ohrvik H, Melo FR, Pejler G. Mefloquine, an anti-malaria agent, causes reactive oxygen species-dependent cell death in mast cells via a secretory granule-mediated pathway. Pharmacol Res Perspect. 2014 Dec;2(6):e00066. doi: 10.1002/prp2.66. Epub 2014 Aug 24. PMID: 25505612; PMCID: PMC4186446. |
| 1. Liu Y, Wong VK, Ko BC, Wong MK, Che CM. Synthesis and cytotoxicity studies of artemisinin derivatives containing lipophilic alkyl carbon chains. Org Lett. 2005 Apr 14;7(8):1561-4. doi: 10.1021/ol050230o. PMID: 15816752. |
| 1. Jayaprakash S, Iso Y, Wan B, Franzblau SG, Kozikowski AP. Design, synthesis, and SAR studies of mefloquine-based ligands as potential antituberculosis agents. ChemMedChem. 2006;1(6):593–7. |
| 1. Trivedi P, Chaturvedi V. Interactive effect of oral anti-hyperglycaemic or anti-hypertensive drugs on the inhibitory and bactericidal activity of first line anti-TB drugs against M. tuberculosis. PLoS One. 2023 Nov 30;18(11):e0292397. doi: 10.1371/journal.pone.0292397. PMID: 38032920; PMCID: PMC10688676. |
| 1. de Sena LWP, Mello AGNC, Ferreira MVD, de Ataide MA, Dias RM, Vieira JLF. Doses of chloroquine in the treatment of malaria by Plasmodium vivax in patients between 2 and 14 years of age from the Brazilian Amazon basin. Malar J. 2019 Dec 21;18(1):439. doi: 10.1186/s12936-019-3072-8. PMID: 31864358; PMCID: PMC6925880. |
| 1. Augustijns P, Geusens P, Verbeke N. Chloroquine levels in blood during chronic treatment of patients with rheumatoid arthritis. Eur J Clin Pharmacol. 1992;42(4):429-33. doi: 10.1007/BF00280130. PMID: 1307690. |
| 1. Todd GD, Hopperus Buma AP, Green MD, Jaspers CA, Lobel HO. Comparison of whole blood and serum levels of mefloquine and its carboxylic acid metabolite. Am J Trop Med Hyg. 1997 Oct;57(4):399-402. doi: 10.4269/ajtmh.1997.57.399. PMID: 9347952. |
| 1. Birgersson S, Van Toi P, Truong NT, Dung NT, Ashton M, Hien TT, Abelö A, Tarning J. Population pharmacokinetic properties of artemisinin in healthy male Vietnamese volunteers. Malar J. 2016 Feb 16;15:90. doi: 10.1186/s12936-016-1134-8. PMID: 26879816; PMCID: PMC4754918. |
| 1. Pasipanodya JG, McIlleron H, Burger A, Wash PA, Smith P, Gumbo T. Serum drug concentrations predictive of pulmonary tuberculosis outcomes. J Infect Dis. 2013 Nov 1;208(9):1464-73. doi: 10.1093/infdis/jit352. Epub 2013 Jul 29. PMID: 23901086; PMCID: PMC3789573. |

Articles identified from:

Reference searching (n =86)

Extra references^*^ (n = 54)

Articles included via database

(n = 13)

Articles included via other methods

(n = 6)

**Total included articles**

**(n = 19)**

Articles excluded based on full text:

Chemical compounds (n = 10)

Not quinolines or artemisinins

(n=4)

Focus not on MTC (n = 29)**

Articles removed before screening

No abstract/full text (n = 220)

Articles identified from PubMed

(n =1187)

Articles assessed for eligibility

(n = 56)

Articles sought for retrieval

(n = 62)

Articles screened

(n =967)

Articles excluded by title + abstract

(n = 905)

No full text available

(n = 6)

Articles sought for retrieval

(n = 8)

Articles assessed for eligibility

(n = 8)

Articles screened

(n =58)

Articles excluded by title + abstract

(n = 50)

No full text available

(n = 0)

Articles removed before screening

Duplicates (n = 80)

No abstract/full text (n =2)

Articles excluded based on full text:

Focus not on MTC (n = 2)**

**Figure S1 Flow Chart of the Literature Review Process**

This diagram outlines the systematic approach taken to review the literature, including the identification and screening of references, and the selection process for inclusion. * Extra references comprise those from initial screening as well as those from the final selected articles. **Exclusions are noted for studies not yielding quantitative or qualitative *in-vitro* results on the growth of the *Mycobacterium tuberculosis* complex (MTC), studies not examining synergistic effects, and those not involving *in-vivo* animal studies or human clinical trials.
